# Editorial: Exploring adverse drug reactions, medication adherence, and forensic markers in pediatrics and obstetrics

**DOI:** 10.3389/fphar.2026.1884873

**Published:** 2026-06-02

**Authors:** Margherita Neri, Angelo Montana, Letizia Alfieri, Pantaleo Greco

**Affiliations:** 1 Department of Medical Science, University of Ferrara, Section of Legal Medicine, Ferrara, Italy; 2 Department of Biomedical Sciences and Public Health, Faculty of Medicine and Surgery, Marche Polytechnic University, Ancona, Italy; 3 Unit of Legal Medicine, AUSL Ferrara, Ferrara, Italy; 4 Gynecology and Obstetrics, University of Salento, Lecce, Italy

**Keywords:** adverse drug reactions (ADRs), forensic markers, forensic pathology, medical malpractice, medication adherence, medication management, pathological outcomes, vulnerable populations

## Introduction

This Research Topic, “Exploring Adverse Drug Reactions, Medication Adherence, and Forensic Markers in Pediatrics and Obstetrics”, emphasizes the essential role of multidisciplinary approaches in addressing contemporary therapeutic challenges, which include evidence on adverse drug reactions, medication adherence, and forensic markers. This special issue also calls for improved surveillance, age-appropriate dosing, adherence strategies, and multidisciplinary forensic approaches. This special issue brings together studies of experimental models and clinical research collected in 17 articles. This editorial frames three interlinked priorities: understanding and preventing adverse drug reactions (ADRs) in vulnerable populations, improving medication adherence to reduce harm and waste, and developing forensic markers to support diagnosis and medicolegal investigations in pediatrics and obstetrics. It emphasizes that developmental physiology, off-label prescribing, and pregnancy-specific pharmacokinetics increase both ADR risk and diagnostic complexity. ADRs and medication adherence are vital to healthcare, especially in pediatric and obstetric care. Children and pregnant women are more susceptible to ADRs, emphasizing the need for careful medication management and precise medical practices. Forensic pathology is crucial for investigating medical malpractice by analyzing medication errors, adherence problems, and the pathological effects of adverse reactions.

## Adverse drug reactions (ADRs)

ADRs in children and pregnant individuals are often underreported and understudied; this collection includes original research, case reports, and pharmacovigilance analyses illustrating serious but preventable harms. In their review and original research articles, the authors explore the effect of preoperative intranasal dexmedetomidine on intraoperative shivering in parturients undergoing cesarean section (Xu et al.), and performed a systematic review and meta-analysis of randomized controlled trials about side effects of intravenously versus intramuscularly oxytocin for postpartum hemorrhage (Ai et al.). In the pediatric field, the paper studied the data from the PharmacoVigilance Center on antiepileptic drugs and fetal disorders (Ji et al.). Clinicians should be vigilant for atypical ADR presentations in neonates and pregnant patients and consider therapeutic drug monitoring where available. Regarding cesarean delivery, the use of ketamine and esketamine is constrained by their dose-dependent side effects. In a meta-analysis, Wei-long Wang et al. accurately quantified the dose-response relationships for preventing shivering and pruritus and assessed the risk of neuropsychiatric side effects, establishing their therapeutic window.

## Medication adherence

Medication adherence is presented in this special issue as a driver of clinical outcomes and pharmaceutical waste; perspectives in the collection linked nonadherence to avoidable ADRs and system costs. An important aspect that was studied was the analysis of off-label drug use and its influencing factors in pediatric otolaryngology and head and neck surgery patients (Ji et al.). In a study, Marano M. et al. assessed the use of esophagogastroduodenoscopy for pediatric gastrointestinal decontamination by retrospectively reviewing cases of suspected or confirmed toxic ingestion in which esophagogastroduodenoscopy was used as the decontamination method.

Combining remimazolam and esketamine effectively was found to reduce adverse hemodynamic effects, such as tachycardia and hypertension, during intubation. However, the optimal dosages for co-administration with remifentanil to facilitate intubation are still unknown. This study aimed to identify the effective doses of remimazolam and esketamine for endotracheal intubation without muscle relaxants in pediatric patients, utilizing Dixon’s up-and-down method (Chen et al.).

This Research Topic highlighted the need for validated concentration reference ranges and biomarkers (e.g., for toxic exposures or therapeutic drug monitoring) to inform clinical and legal decisions in neonates, children, and parturients. Recommendations and research priorities focused on strengthening pharmacovigilance through age- and pregnancy-specific reporting and active surveillance systems and the integration of adherence interventions that combine policy (e.g., waste reduction and prescribing practices) with patient-level supports (e.g., education and formulation changes, such as minitablets). Policymakers must balance access to necessary medicines with stricter monitoring to reduce off-label risks and pharmaceutical waste.

This collection of studies calls for coordinated action across clinical care, research, and forensic practice to reduce ADRs, improve adherence, and develop reliable forensic markers in pediatrics and obstetrics. It serves as both a synthesis of recent studies and a roadmap for targeted research and policy interventions.

## Conclusion

Medication adherence and forensic markers in pediatric and obstetric care are essential for enhancing patient safety and clinical results when addressing adverse drug reactions. Gaining insights and reducing the risks associated with medication use in these vulnerable groups helps healthcare providers achieve better treatment outcomes and comply with legal safeguards. This collection aims to guide future research, clinical practice, and policy development to promote a safer and more efficient healthcare system.

